# Humble Leadership Benefits Employee Job Performance: The Role of Supervisor–Subordinate *Guanxi* and Perceived Leader Integrity

**DOI:** 10.3389/fpsyg.2022.936842

**Published:** 2022-07-05

**Authors:** Bin Yang, Yimo Shen, Chenlu Ma

**Affiliations:** ^1^Faculty of Psychology, Southwest University, Chongqing, China; ^2^Faculty of Psychology, Beijing Normal University, Beijing, China

**Keywords:** humble leadership, job performance, perceived leader integrity, supervisor–subordinate *guanxi*, social exchange theory, attribution theory

## Abstract

Although humility is a hallmark of many beloved and respected leaders, yet little is known about the impact of humble leadership on employee job performance. Drawing on social exchange theory and attribution theory, the current study suggests a moderated mediation model to elucidate how and when humble leadership encourages follower job performance. Analyses of multilevel, multisource data from 204 subordinates and 68 supervisors showed that humble leadership and employee job performance *via* supervisor–subordinate *guanxi* is moderated by perceived leader integrity, such that the indirect and positive relationship between humble leadership and employee job performance *via* supervisor–subordinate *guanxi* would be strengthened when perceived leader integrity is high rather than low. Theoretical and practical implications as well as limitations and future directions are discussed.

## Introduction

The scandal of Enron caused by leader’s hubris, uncontrolled ego and entitlement has drawn leadership scholars’ attention to the value of leader humility (Owens et al., 2013). On the one hand, leaders who are over confident, narcissistic and arrogant often overlook other’s ideas and feedbacks, bringing about mistakes and scandals (Kelemen et al., 2022). On the other hand, leadership scholarship recently emphasizes leaders’ role in facilitating employee growth and full potential achievement (Cho et al., 2020). Thereby, humble leadership, as an effective and promising leadership style, has received increasing interests from both scholars and practitioners (e.g., Owens and Hekman, 2012; Ou et al., 2014; Owens et al., 2016; Hu et al., 2018). Humility is an integrative part of organizational virtues and is vital for leaders to lead in environments full of unpredictability and turbulence (Vera and Rodriguez-Lopez, 2004). Humble leadership has been documented to contribute to a wide range of positive consequences, including increases in employee voice behavior (Bharanitharan et al., 2018; Li et al., 2018; Ma et al., 2019), proactive behavior (Chen et al., 2018; Lu et al., 2018), feedback-seeking behavior (Qian et al., 2020), gratitude (Naseer et al., 2020), self-efficacy (Mao et al., 2018), well-being (Zhang and Song, 2020), among various others (for an elaborative review, see Kelemen et al., 2022).

Despite the proliferation of humble leadership studies, research on this area is still premature and in infancy (Qin et al., 2020). One scarcity lies in the exploration of the underlying processes that humble leadership influences important positive outcomes and the boundary conditions in which humble leadership is more or less effective (Carnevale et al., 2019; Cho et al., 2020; Kelemen et al., 2022). Investigating mediation mechanisms is crucial to the theoretical advancement of the humble leadership literature because it makes it possible to reveal a larger nominal network of this leadership style. Based on previous insights, we aim to gain a better understanding of how humble leadership influences employee job performance. We identify job performance as dependent variable because it is crucial for organization’s overall functioning (e.g., Hui et al., 1999). Although a handful of research showed that humble leadership cast positive impacts on employee job performance (e.g., Wang et al., 2018; Cho et al., 2020; Bahadur and Ali, 2021), yet little is known about psychological mechanism underlying how employees interpret the effects of humble leadership at the individual level, resulting in improved job performance (Diao et al., 2019). In such a case, the current study investigates the underestimated relationship between humble leadership and subordinate job performance.

Previous research indicates that the process of leadership’s influence on employees’ attitudes and behaviors can be seen as a social exchange process between leader-employee dyads based on the principle of reciprocity (Lam et al., 2007; Chan and Mak, 2012). Accordingly, we refer to social exchange theory as our theoretical foundation. Social exchange theory posits that one party do a favor for the other party with the assumption that they will receive unspecified returns in the future (Blau, 1964). Social behavior is the exchange of valuable material or non-material things, such as feelings of honor or prestige (Homans, 1958). Considering that leader humility is deemed as relational in nature (Owens et al., 2011) and serves as a relationship builder (Nielsen et al., 2013), we propose that supervisor-subordinate *guanxi* (SSG) might act as a mediator that accounts for the exchange effect between humble leadership and job performance. SSG is a kind of dyadic, specific, and emotive tie that has the ability to facilitate positive interactions between the parties linked by the tie (Bian, 2006, p. 312), perfectly capturing the essence of social exchange. Humble leaders shine the spotlight on their employees rather than themselves, pinpoint their followers’ strength and contributions, and emphasize employee learning and growth, which may at the outset earn employees’ liking and trust (Nielsen et al., 2010), laying the foundation for building high-quality SSG. And high-quality SSG is predisposed to stimulate employee job performance, for employees will repay the goodwill received from humble leaders by improving their job performance. Thus, we surmise that SSG will be the mediating factor that links humble leadership and job performance, based on social exchange theory.

Further, researchers suggest that the effectiveness of humble leadership pivots not only on the expressed humility behaviors, but also on how employees attribute such behaviors (Nielsen et al., 2010; Owens and Hekman, 2012; Qin et al., 2020). Attribution theory states that people are intrinsically inclined to decipher others’ acts in terms of their causes in order to make sense of their surroundings (Kelley and Michela, 1980; Martinko et al., 2007). Owens and Hekman (2012) suggest that when leader display humility behaviors, employees will be positively motivated if they perceive such actions as authentic and sincere. But if they perceive their leader humility as false and instrumental, they will adopt a defensive mindset and show distrust and disrespect for their leaders. Leader integrity implies that the leader has morality and his or her moral behaviors are consistent over time and across situations (Moorman et al., 2012). Given that leader humility is more likely to be perceived as genuine when employees observe leaders exhibiting integrity at the same time, we draw on attribution theory (Kelley and Michela, 1980; Martinko et al., 2007), and identify perceived leader integrity as a contingency that strengthens the effectiveness of humble leadership.

Our study contributes to the literature in several ways. Firstly, drawing on social exchange theory, we find that humble leadership contributes to SSG, which in turn, promotes follower job performance. Yang (1992) states that in the Chinese context, supervisor–subordinate relationships can shift from a contract-based economic exchange mode to a communal sharing one through familiarization process. We give a clue that when interact with humble leaders, employees are more likely to experience such kind of supervisor–subordinate relationship, which in turn, stimulates followers to augment their job performance. Secondly, we draw a more complete picture by verifying the conditional effect of leader integrity. As sincerity-based trust carries a heavier weight in developing close *guanxi* than ability-based trust (Chen and Chen, 2004), we include integrity as our moderator. The consistence between leader’s words and deeds could increase follower’s faith in their leader’s sincerity, and thus strengthen the positive effect of humble leadership. Thirdly, we extend the literature pertaining to SSG by implying that the perception of humble leader’s trustworthiness and the positive feelings derived from them promotes SSG. Guanxi emphasizes adhering more firmly to one’s work role commitments (Chen and Chen, 2009), yet the antecedents of SSG or guanxi in general have received very little attention (Zhang et al., 2014). Humble leaders signal that their employees’ contributions are valued and their mistakes are tolerable, beneficial for the enactment of SSG.

## Theoretical Background and Hypothesis Development

### The Relationship Between Humble Leadership and Job Performance

Humble leadership refers to leader’s expressed humility in interpersonal interaction and such humility is observable and comprises three key dimensions: (a) a manifested willingness to view oneself accurately (i.e., showing a willingness to evaluate oneself without positive or negative exaggeration), (b) a displayed appreciation of others’ strengths and contributions (i.e., showing appreciation for the unique strengths and contributions of others), and (c) modeling teachability (i.e., showing openness to new ideas, feedback, and advice; Owens et al., 2013; Rego et al., 2017).

As humble leaders focus on employee growth and development, we suggest such kind behavior would invoke a positive exchange from employees. Evidences indicate that employees will reciprocate their leaders by exhibiting more beneficial behaviors and inhibiting counterproductive behaviors when they perceive they are fairly treated and their leaders are trustworthy (Sousa-Lima et al., 2013; Wang et al., 2019). Improving job performance is a way to repay leader’s goodwill and kindness (Chen and Yang, 2012; Liu and Wang, 2013). Humble leaders enhance followers affective trust by sharing and delegating control, facilitating open communication, and demonstrating care and support for their employees (Liborius and Kiewitz, 2022). Besides, humble leaders applaud employees’ success rather than taking credit from their followers (Morris et al., 2005; Wang et al., 2018), increasing their followers’ fairness perception. Consequently, employees will feel an obligation to give something kind and desirable back to their leaders. As leader performance is oftentimes rely on employee performance, employee might work harder to create more value for their supervisors. Therefore, we propose:

*Hypothesis 1*: Humble leadership is positively related with employee job performance.

### The Mediating Role of SSG

In addition, we expect that the relationship between humble leadership and job performance is mediated by SSG, which is defined as “a dyadic, particular, and sentimental tie that has the potential of facilitating favor exchanges between the person and his/her immediate supervisor connected by the tie” (Zhang et al., 2014). SSG can be differentiated from leader-member exchange (LMX) in threefold. Firstly, LMX is developed through work-related activities and restricted to work-related exchanges, while SSG is built up through non-work-related activities and involves both instrumental and emotional exchanges (Zhang et al., 2017). Secondly, LMX emphasizes equity rules during the exchange process, yet SSG initiates exchanges according to different roles in the particularistic relationship (Chen and Chen, 2009). Thirdly, LMX puts job as the top priority, whereas SSG prioritizes relationships (Zhang et al., 2014). Although both LMX and SSG are grounded in social exchange theory, we chose SSG as the mediator for two reasons. In the first place, past studies suggest that SSG is more strongly associated with person-supervisor fit which will result in behaviors targeting the supervisor, while LMX better predicts person-organization fit and contributes to behaviors relating to organization (Zhang et al., 2017). Secondly, in the Chinese context, people are so relation based that they pursue *guanxi* for its own sake (Chen et al., 2013), reflecting the characteristic of SSG rather than LMX.

Social exchange theory (Blau, 1964) identifies two forms of exchange relationships, namely economic exchange and social exchange. Social exchange will beget personal obligation, trust and gratitude (Blau, 1964), resulting in the reciprocal behaviors from organizational members. Grounded in social exchange theory, we predict humble leadership will augment SSG, which in turn, promotes job performance.

SSG is comprised of five main components: ganqing (affect), renqing (reciprocal exchange of favor), face, personal life inclusion and trust (Zhai et al., 2013). We argue that humble leadership could cast a positive effect on all these aspects. Firstly, humble leaders evaluate themselves objectively and accurately without any denial of applaudable achievements and/or self-defense against criticisms, which will increase their attraction to their employees because they do not defensively react to others’ critiques and wisely absorb constructive information (Ryan, 1983). Accordingly, employee will generate positive affection toward their supervisors. Secondly, humble leaders are “other-oriented,” manifested by the embracement of others into their own self-concept and the intention to help others develop and grow (Ou et al., 2014; Wang et al., 2018). Humble leaders will give employees job autonomy, inspire them to make the most of their strengths and cheer for their accomplishments (Owens and Hekman, 2012). These behaviors could be taken as supervisor-initiated favors, engendering a feeling of renqing. Thirdly, humble leaders’ displaying teachability signals an attitude of tolerating errors and legitimizing uncertainties (Owens and Hekman, 2012). When employees make mistakes, chances are that they will not be humiliated or despised by their supervisors, saving employee’s lian. Meanwhile, employees working with humble leaders feel they are valued and respected by their leaders and perceive themselves as organization insiders (Owens and Hekman, 2012; Zhu et al., 2019), increasing their feeling of mianzi. Thereafter, employees will beget a feeling of honor when lian and mianzi, two components of face, are cultivated. Fourthly, leaders who are humble display transparent, friendly attitudes, seek guidance and listen to how followers feel about them, narrowing the power distance between supervisors and subordinates (Jeung and Yoon, 2018). It would be easier for employees to interact with supervisors outside the workplace when power distance is at low level. Lastly, employees are paradoxical in that they will engender a feeling of unfairness when *guanxi* becomes the benchmark for supervisor’s decision-making while enjoying the favors from close *guanxi* relations with their leaders (Chen et al., 2011). The follower-oriented quality of humble leaders will be in a strong position to eliminate employees’ worries about the possible nepotism and leaders’ taking advantage of personal relationships to pursue individual benefits or engage in under the table transactions, facilitating employees’ trust in their supervisors. Following the growth of trust, SSG will be naturally increased. In a nutshell, leader humility facilitates the high quality of SSG through promoting SSG’s five main parts.

Ensuing the high quality of SSG, the supervisor-initiated social exchange creates a felt obligation on the part of organizational members to reciprocate their leaders’ trust and liking through positive ways, such as working hard to meet their leaders’ expectations (Settoon et al., 1996; Gerstner and Day, 1997). Consequently, employees will exert more efforts when dealing with their jobs, in order to repay the gained goodwill and maintain the long-term development of the relationship. Combing these arguments, we propose the following hypothesis:

*Hypothesis 2*: SSG mediates the relationship between humble leadership and job performance.

### The Moderating Role of Perceived Leader Integrity

Although we anticipate that humble leadership will generally benefit SSG, drawing on attribution theory (Kelley and Michela, 1980; Weiner, 1985), we further suggest that such effect will be bounded by perceived leader integrity. Humble leadership does not always prompt positive outcomes, as it is the case that abusive supervision does not uniformly lead to negative consequences. The outcomes vary because employee attribute these objective behaviors distinctly (Qin et al., 2020; Yu and Duffy, 2021). As demonstrated by previous researchers, employees’ interpretations of other’s motives will determine how these behaviors influence their subsequent responses and the relationships with others (Lam et al., 2007; Carnevale et al., 2019; Gardner et al., 2019; Qin et al., 2020). Owens et al.’s (2012) qualitative study revealed that leaders’ behaviors of highlighting employees’ strengths and contributions are only effective when perceived as sincere and non-instrumental, otherwise followers will distrust and contempt their leaders.

According to attribution theory, people are intrinsically inclined to understand others’ acts in terms of their causes, trying to make sense of the world around them (Kelley and Michela, 1980; Weiner, 1985). When leaders display humility toward followers, followers might wonder why they treated them like that. If the relevant evidence strongly and consistently points to a specific cause for the action, making an attribution should be relatively simple (Hamilton et al., 1990). Therefore, we suggest that perceived leader integrity acts as a circumstance to aid the inference process of leader humility in that it provides consistent information across situations.

Leader integrity refers to “a multidimensional construct capturing both perceptions that the leader holds moral values and professes as well as enacts those values with an exceedingly high degree of consistency” (Moorman et al., 2013). A leader of integrity would behave morally and consistently over time and across situations. Researchers pointed out that leaders with high integrity are less likely to be self-serving and more likely to be concerned with followers’ interests rather than the interests of their own, compared with leaders with low integrity (Jiang et al., 2014), and thus these conducts are more compatible with humble leaders’ expressed care and altruism, increasing employee’s faith in leader’s genuine humility. Moreover, leaders having integrity are characterized by trustworthiness, fairness, morality, fidelity and honesty (Mayer and Davis, 1999; Moorman et al., 2013; Hewlin et al., 2017). Accordingly, when observing leader’s integrity, followers are equipped with more evidence to believe that their immediate leader is an authentic person who will not fake humility for impression management or other utilitarian reasons. What is more, it is suggested that one of the reasons why employees care about a leader’s integrity, according to scholars, is that it acts as a helpful tool to attenuate confusion in the decision to follow (Moorman and Grover, 2009), as feelings of ambiguity and uncertainty are generally aversive for most of people (Duan et al., 2020; Fiske and Taylor, 2020). Therefore, working with leaders having high integrity predisposes followers to better predict leader’s future deeds from his or her words (Moorman and Grover, 2009), reducing the apprehension of leader’s hypocrisy. In the contrary, a leader who is arbitrary instead of consistent will not be counted on to do the right thing and conduct morally (Bass, 1998). In sum, perceived leader integrity serves as an available information for employees to attribute leaders’ humble behaviors to their internal good character rather than external instrumental factors, which in turn amplifies its positive effect on SSG. But when perceived leader integrity is at low level, employees will have difficulty in inferring the causes of leader humble behaviors, and thus being suspicious of leader’s intentions. Consequently, employee’s affection, trust and honor generated by leader’s humility would be curtailed, reducing the positive effect of humble leadership on SSG. On the basis of the abovementioned descriptions, we propose that:

*Hypothesis 3*: Perceived leader integrity moderates the relationship between humble leadership and SSG, such that the positive relationship will be stronger when perceived leader integrity is relatively high rather than low.

### The Moderated Mediation Model

Combining our hypothesis 1 with hypothesis 2, we continue to propose a moderated mediation model, in which perceived leader integrity moderates the indirect relationship between humble leadership and job performance through the effect of SSG. When leader integrity is perceived as high, the effect of humble leadership on SSG would be optimized, and hence compel employees to hurl themselves into work to fulfill their leaders’ expectations and sustain this high-quality relationship. As a result, the positive indirect effect would be intensified under situations of high perceived leader integrity. Thereafter, we propose:

*Hypothesis 4*: The humble leadership-SSG-job performance relationship will be moderated by perceived leader integrity, such that it will be stronger when leader integrity in at high level rather than low level.

**Figure 1:**
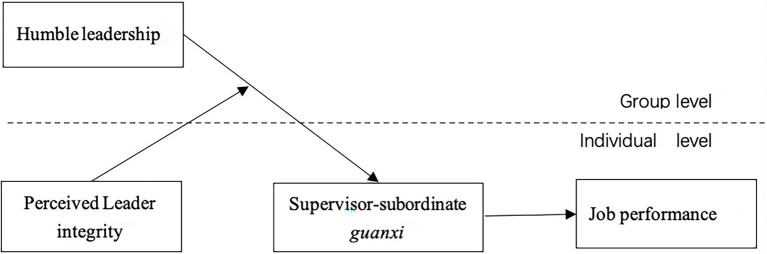
The hypothetical model.

Figure 1 is our hypothetical model.

## Materials and Methods

### Participant and Procedure

Data were collected in 25 Chinese enterprises, engaging in real estate, electronic manufacturing, information technology, education and training industries, among others. During the investigation process, we first negotiated with company’s department of human resource management to explain the purpose and procedures of our study. Supervisors randomly selected employees to participate in the research. We delivered the matching surveys to subordinates and supervisors separately. Followers were asked to respond to humble leadership, leadership integrity, SSG and their demographics. Their immediate leaders were responsible for evaluating follower’s job performance. Completed questionnaires in sealed envelopes were directly returned to investigators on the spot. All subjects received a small gift as a token of our gratitude for their voluntary participation. Confidentiality of the study was informed and ensured. Among 410 distributed paired surveys, 204 dyadic questionnaires were confirmed as valid, filtering out 206 questionnaires having less than three employees nested in the same group or leaving many key variables missing. 49.8% of the final sample were women; 80.4% aged from 20 to 30 years old; 48% got a degree of bachelor or above. The average employee tenure was 5.08 years.

### Measures

#### Humble Leadership

We adopted a nine-item scale developed by Owens et al. (2013) to measure humble leadership. Sample items are “My leader often compliments others on their strengths,” and “My leader shows appreciation for the unique contributions of others.” Previous studies suggested that leadership can be considered as an individual or a group-level phenomenon (e.g., Liao and Chuang, 2007; Mayer et al., 2012). Accordingly, we calculated the within-group consistency and between-group variances to decide whether it is appropriate to aggregate the individual’s humble leadership into the group level. Results provide sufficient justification for aggregating humble leadership, with ICC(1) = 0.43, ICC(2) = 0.70, and the medium and the mean R*_wg(j)_* were 0.98 and 0.97, respectively. Therefore, we investigate humble leadership at the team level and use the mean of all respondents from each group to index humble leadership. The reliability of the scale in the current study was 0.93.

#### Perceived Leader Integrity

Followers reported perceived leader integrity using 8 items from Simons et al. (2007). Sample items include, “When my manager promises something, I can be certain that it will happen,” “My manager practices what he/she preaches,” and “There is a match between my manager’s words and actions.” The reliability of the scale in the current study was 0.92.

#### Supervisor–Subordinate Guanxi

Employees self-reported SSG using the six-item scale developed by Law et al. (2000). Example items include, “During holidays or after office hours, 1 would call my supervisor or visit him/her,” “On special occasions such as my supervisor’s birthday, I would definitely visit my supervisor and send him/her gifts.” The reliability of the scale in the current study was 0.79.

#### Job Performance

The immediate supervisor evaluated their follower’s performance based on four items from Farh and Cheng (1997). A sample item is “This person is one of the best employees in our work unit.” The reliability of the scale in the current study was 0.72.

#### Control Variables

Because employees’ demographic factors may affect their attitudes as well as their performance (Wang et al., 2018), we controlled for follower’s gender, education, age, and tenure in the analyses. Research found that gender correlates with leadership perceptions and performance outcomes (Riaz et al., 2018) and age is positively related to job performance (Yang et al., 2017). Employees with higher degrees or longer tenure are prone to perform better (Maslyn and Uhl-Bien, 2001; Ng and Feldman, 2010). All focal variables’ items were rated on five-point Likert scale (1 = strongly disagree, 2 = disagree, 3 = neutral, 4 = agree, 5 = strongly agree). All the scales originally written in English (i.e., humble leadership and leader integrity) were translated into Chinese before data collection through the procedure of translation and back-translation to warrant the conceptual equivalence (Brislin, 1980).

### Analytical Strategy

Before we test our hypotheses, a series of confirmatory factor analysis (CFA) was first applied to confirm the validity of our focal variables. We then tested the path coefficients in the multilevel model using Mplus 8.0 (Muthén and Muthén, 2017) to verify our hypotheses. Considering the nested nature of our data, we grand-mean centered humble leadership and group-mean centered perceived leader integrity when testing the moderating effect of perceived leader integrity. When it comes to the indirect effects in multilevel analyses, we adopted Monte Carlo resampling method to improve the accuracy (Bauer et al., 2006). Preacher and Selig (2012) pointed out that the Monte Carlo method outperforms the Sobel test, which computes confidence intervals based on a single sample of data. Twenty thousand resamplings were used to compute each confidence interval.

## Results

### Confirmatory Factor Analysis

Before testing our model, we used LISREL8.7 to execute the confirmatory factor analysis (CFA), in order to examine the discriminant and convergent validity of our key factors (i.e., humble leadership, SSG, perceived leader integrity and job performance). CFA results shows that the factor loading of the baseline four-factor model have reached a significant level of 0.05, and there is no improper solution, indicating that the four constructs involved in this study have good convergent validity. Further, results in Table 1 demonstrated that our four-factor model has superior fit index than other five competitive models (χ^2^/*df* = 2.46, RMSEA =0.085, CFI = 0.96, NFI = 0.93). Therefore, we are convinced that our key variables are different variables.

**Table 1:** Results of the confirmatory factor analysis.

Model	Factors	χ^2^	*df*	χ^2^/df	RMSEA	CFI	NFI	Model comparison test		
								Comprison	Δχ^2^	Δ*df*
Model 1: the baseline model	Four factors	846.38	344	2.46	0.085	0.96	0.93			
Model 2	Three factors; based on model 1, humble leadership and leader integrity were combined into 1 factor	1492.88	347	4.30	0.128	0.93	0.91	2 vs.1	646.5^**^	3
Model3	Three factors; based on model 1, leader integrity and SSG were combined into 1 factor	1160.92	347	3.35	0.107	0.94	0.92	3 vs.1	314.54^**^	3
Model4	Three factors; based on model 1, humble leadership and SSG were combined into 1 factor	1021.73	347	2.94	0.098	0.95	0.92	4 vs.1	175.35^**^	3
Model 5	One factor; all four factors were combined into one factor	1802.45	350	5.15	0.143	0.91	0.89	5 vs.1	956.07^**^	6

*N* = 204. NFI, non-normed fit index; CFI, the comparative fit index; and RMSEA, the root-mean-square error of approximation.

** *p* < 0.01 (two-tailed).

### Descriptive Statistics

The means, standard deviations, and correlations among the study variables are shown in Table 2. As presented in Table 2, SSG is positively associated with job performance (*r* = 0.50, *p* < 0.01), in line with our predictions.

**Table 2:** Means, standard deviations, and correlations between variables.

Variable	*M*	*SD*	1	2	3	4	5	6	7
Individual level									
1. Subordinate’s gender	1.50	0.50	--						
2. Subordinate’s age	27.35	5.70	0.03	--					
3.Subordinate’s education	3.33	0.75	0.09	−0.05	--				
4. Subordinate’s tenure	5.08	4.54	−0.00	0.75^**^	−0.20^**^	--			
5. Perceived leader integrity	3.59	0.78	0.07	0.06	−0.15^*^	0.13	**(0.92)**		
6. SSG	3.16	0.71	0.01	−0.03	0.07	−0.01	0.47^**^	**(0.72)**	
7. Job performance	3.76	0.71	0.17^*^	−0.05	0.06	0.02	0.48^**^	0.50^**^	**(0.79)**
Group level									
1. Humble leadership	3.67	0.59	(0.93)						

*N* = 204 at level 1. *N* = 68 at level 2. Reliabilities of the scales are boldfaced and noted in the diagonals. Gender had two categories: 1 = male, 2 = female. Education had four categories: 1 = middle school education, 2 = high-school education, 3 = specialty education, 4 = undergraduate education or above.

* *p* < 0.05;

** *p* < 0.01 (two-tailed).

### Hypothesis Testing

Hypothesis 1 expected a positive relationship between humble leadership and job performance. The regression coefficient is nonsignificant (*β* = 0.18, *p* > 0.05), which is not supportive of hypothesis 1.

Hypothesis 2 postulated that the effect of humble leadership on job performance is transmitted through SSG. As we can see from Table 4, the indirect effect is 0.28, with a confidence interval not including zero (95% C.I. = [0.15, 0.42]), supporting the mediating effect of SSG. Thus, hypothesis 2 is supported.

**Figure 2:**
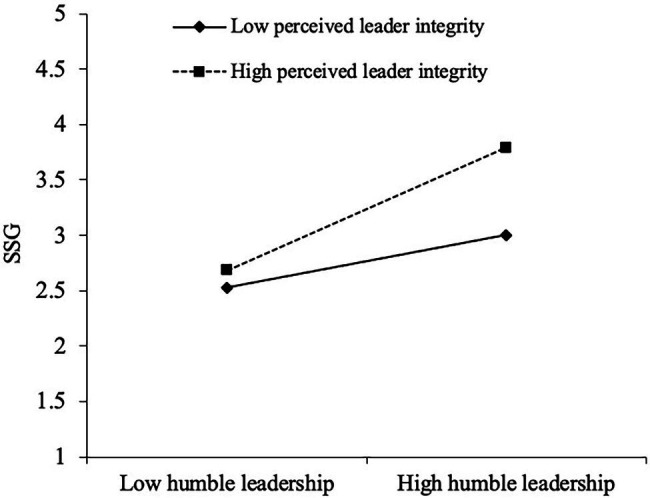
The moderating effect of leader integrity.

In hypothesis 3, we assumed that perceived leader integrity moderates the relationship between humble leadership and job performance. Path-analytic regression has been adopted to verify this hypothesis. As presented in Table 3, the interaction of humble leadership and perceived leader integrity is positively related with SSG (*β* = 0.34*, p* < 0.05) after controlling the main effect of humble leadership and leader integrity. To further explore whether the interaction pattern is consistent with our proposition, we plotted the interaction following Aiken and West’s (1991) advice of computing simple slopes. Figure 2 illustrates that the positive relation between humble leadership and SSG is stronger for those respondents who perceived their leader as high in integrity than those who perceived their leader as low in integrity (when perceived leader integrity is one standard deviation above the mean: simple slope = 0.94, *p* < 0.001; when perceived leader integrity is one standard deviation below the mean: simple slope = 0.40, *p* < 0.001). The difference between the two is significant (Δγ = 0.54, *p* < 0.05). As a result, hypothesis 3 is supported.

**Table 3:** The moderating effect of leader integrity.

Variable	SSG		Job performance	
	Model 1	Model 1	Model 2	Model 2
Intercept individual level	0.10^**^(0.54)	2.69^**^(0.34)	1.00 (0.55)	3.43^**^(0.35)
Gender	−0.11 (0.08)	−0.12 (0.06)	0.10 (0.09)	0.09 (0.08)
Age	0.01 (0.01)	0.01 (0.01)	−0.01 (0.02)	−0.01 (0.02)
Education	0.13 (0.06)	0.12 (0.06)	0.11 (0.09)	0.10 (0.09)
Tenure	−0.02 (0.02)	−0.02 (0.02)	0.00(0.02)	0.00(0.02)
Perceived leader integrity	0.27^**^ (0.09)	0.30^**^ (0.08)	0.43^**^ (0.06)	0.45^**^ (0.07)
Humble leadership ×perceived leader integrity		0.34^*^(0.15)		0.23 (0.14)
Group level				
Humble leadership	0.45^**^ (0.13)	0.67^**^ (0.09)	0.18 (0.13)	0.25** (0.09)

*N* = 204 at level 1. *N* = 68 at level 2. Entries are estimates of effects with standard errors.

* *p* < 0.05;

** *p* < 0.01 (two-tailed).

Table 4 exhibits the results of our moderated mediation model testing. We can find that when perceived leader integrity is high, the indirect relationship between humble leadership on job performance *via* SSG is stronger (indirect effect = 0.39, 95% C.I. = [0.20, 0.62]), compared to the condition where perceived leader integrity is low (indirect effect = 0.17, 95% C.I. = [0.05, 0.30]). The difference between the two levels is at significant level (Δγ = 0.22, 95% C.I. = [0.03, 0.46]). Thereafter, hypothesis 4 is supported.

**Table 4:** Conditional indirect effects of humble leadership on job performance *via* SSG.

Moderator: perceived leader integrity	Humble leadership (X) ➔ SSG (M) ➔ job performance (Y)			
	Stage		Effect	95% C.I. of indirect effect
	First (*P*_MX_)	Second (*P*_YM_)	Indirect (*P*_MX_ *P*_YM_)	
Low (−1*SD*)	0.40^**^(0.13)	0.41^**^(0.08)	0.17^*^(0.07)	[0.05, 0.30]
*M*	0.67^**^(0.09)	0.41^**^(0.08)	0.28^**^ (0.07) 0.39^**^ (0.11) 0.22^*^(0.11)	[0.15, 0.42]
High (+1*SD*)	0.94^**^(0.16)	0.41^**^(0.08)		[0.20, 0.62]
Difference between low and high	0.53^*^(0.23)	0.41^**^(0.08)		[0.03 0.46]

*N* = 204 at level 1. *N* = 68 at level 2. Entries are estimates of fixed effects with standard errors. P_MX_ refers to path from humble leadership to SSG; P_YM_ refers to paths from SSG to job performance. C.I., confidence interval. +/−1 *SD* distinguishes higher from lower levels of perceived leader integrity.

The coefficient for each conditional indirect effect was estimated using the product of coefficients approach.

Confidence intervals were calculated using the Monte Carlo method. Parameters estimated in separate SEM analyses were input to the utility provided by Selig and Preacher (2008; http://quantpsy.org/medmc/medmc.htm).

* *p* < 0.05;

** *p* < 0.01 (two-tailed).

## Discussion

Through our empirical study, we find evidence for the mediating effect of SSG in the relationship between humble leadership and job performance. And we also discover that when having a leader high in integrity, employees would be more willing to build SSG of high quality, facilitating the enhancement of their job performance, whereas having a leader low in integrity, employees’ tendency to develop SSG is weakened, which in turn influences their subsequent job performance.

### Theoretical Implication

Our study carries several important theoretical implications. Firstly, we expand the nomological network of humble leadership by identifying a new mediator. Given the positive outcomes humble leaders bringing about, more academic rigor has been added to the research on humble leadership (e.g., Rego et al., 2017; Chen et al., 2018; Mao et al., 2018; Ma et al., 2019; Zhang and Song, 2020). However, no research to date has considered the *guanxi* impact of humble leadership, of which humble leaders can take advantage to affect employee job performance. This is an omission because *guanxi* in Chinese society is ubiquitous and becomes one of the principles guiding behaviors (Chen et al., 2013). By applying the underlying logic of social exchange theory (Blau, 1964), we propose and test the possible mediating role of SSG. Humble leader’s behaviors, such as viewing themselves accurately, admiring and appreciating employee’s merits and contributions (Owens and Hekman, 2012; Owens et al., 2013), enhance employee’s felt respect, trust and consideration and protect follower’s “face,” giving rise to high-quality SSG. SSG, in turn, fuels employees to improve their job performance because of the principle of “reciprocity.” The results contribute to enriching the literature of humble leadership and social exchange theory.

Secondly, we enrich our knowledge about the boundary context of humble leadership. The main cause of variation in follower reactions is followers’ attributions and perceptions (Moorman and Grover, 2009). Thus, drawing on attribution theory, we evidence that perceived leader integrity can serve as a useful information which employees utilize to make attributions of their leader’s humble behaviors. And such finding is in line with previous qualitative and quantitative research emphasizing leader sincerity in exploiting the utility of leader humility (Owens and Hekman, 2012; Ma et al., 2019; Kelemen et al., 2022). It appears from our study that perceived leader integrity exerts an immense influence on shaping employee’s perception of humble leadership’s sincerity and honesty. A leader with high integrity signifies that he or she has moral ethics and will align deeds to words (Moorman et al., 2012), apparently showing their trustworthiness and goodwill to followers. In this case, employees would be more inclined to form positive *guanxi* with their supervisors, resulting in high job performance.

Finally, we extend the literature pertaining to SSG by establishing humble leadership as its antecedents. As indicated by Chen et al. (2011), the practice of *guanxi* is not always eliciting beneficial consequences but could engender undesirable results, including nepotism, corruption, violation of organizational processes, and a loss of trust in the authority (Bian, 1994; Verhezen, 2008), raising a question of the ethics of SSG. Zhang et al., (2014) argue that investigating *guanxi*‘s antecedents gives insights that may help to overcome the discrepancy between arguments and facts about *guanxi*‘s ethics. However, the exploration of *guanxi*’s antecedents is rare. Identifying humble leadership as its proximal factor gives a clue that when SSG is generated through positive feelings such as like, perceived trust and respect, it is more likely SSG precipitates positive results (e.g., job performance).

### Practical Implications

In addition to these theoretical implications, our study is also of practical significance. Researches have shown that building personal *guanxi* is fundamental for effective leadership (Hui and Graen, 1997). Consistent with this argument, we prove that SSG, resulting from humble leadership, is positively associated with employee job performance. Therefore, it is desirable that managers build personal, outside the work domain relationship with subordinates to stimulate their job performance. Further, because SSG could be built through leader expressed humility, humility in leader should be encouraged. Previous research on humility has revealed that intervention workbooks can improve participants’ perceptions of humility (Lavelock et al., 2017). To aid the cultivation of leader humility, some leader training programs might be fruitful.

However, managers should be cautious about engaging in personal relationships with their followers through humble behaviors. Specifically speaking, accompanied with observable humble behavior, sincerity and honesty are necessitated to yield the effect of humble leadership to the maximum extent. Otherwise, the positive effect might be attenuated as the suspicion and contempt rising from false humility can be detrimental for employee outcomes (Owens and Hekman, 2012). The interaction of humble leadership and perceived leader integrity enlightens us that managers are able to increase followers’ perception of sincerity in displayed humility by consistently and morally conduct their behaviors. In realistic situation, leaders’ characteristics are functioning together rather than in isolation (Derue et al., 2011). Accordingly, our suggestion is that when exhibiting humility, managers should provide supplementary information to help employees to feel high sincerity, like demonstrating high integrity.

### Limitation and Future Direction

Our study is by no means without limitations. Firstly, we collected our data at the same time point, leaving causal inferences unsubstantiated. To reduce the concern that SSG influences employee’s perception of humble leadership, we followed the advice of Kline (2011) to compare the two non-nested models using Akaike’s Informative Criterion (AIC) and Bayesian Information Criterion (BIC). The results demonstrated that our hypothetical model (AIC = 364.69 and BIC = 374.64) had a superior fit than the reverse causal model (AIC = 379.78 and BIC = 389.74). However, we still cannot infer the causal effects among these variables. Thus, future research is encouraged to adopt experimental or longitudinal design to determine the causal effect between our independent variable and its outcomes. Secondly, the same data source (i.e., employee) of humble leadership, leader integrity and SSG raises the concern of common method bias in spite of our efforts to decrease its effect by collecting job performance from a different source (i.e., supervisor; Podsakoff et al., 2012). However, we have reasons to do so. Our study is employee-centric and aims to capture the underlying processes of employees in reaction to humble leadership. Besides, the definition of humble leadership stress that it must be observable (Owens et al., 2013), rendering it pretty easy for employee to make evaluations. Leader integrity comprises of consistency aspect (Moorman et al., 2013), which could also be easily judged by employees. When it comes to SSG, it is quite particularistic that others might have difficulty in appraising it. Correspondingly, we rated humble leadership, SSG and perceived leader integrity from the perspective of employees. But we do admit that employee’s perception of leader behaviors is affected by their characteristics (Wang et al., 2018), hoping future research could adopt more objective ways to add insights in this regard. Finally, our research is limited in the studied sample, restricting the generalizability of the findings. As is known for us, personal *guanxi* in China is embedded in Chinese culture, and thus it may not fit in western context. To tackle this problem, cross-culture investigation would be useful in pinpointing the applicability of our study results.

## Conclusion

Considering the importance of humble leadership, we aimed to test the effects of humble leadership on employee job performance. Samples from 204 employees clustered in 68 groups support that humble leadership has a positive relationship with employee job performance, which is mediated by SSG. We further found that perceived leader integrity acts as a moderator in the indirect relationship between humble leadership and job performance. This study provides some insights on how and when humble leadership affects job performance and hopes to inspire more research dedicated into this area.

## Data Availability Statement

The raw data supporting the conclusions of this article will be made available by the authors, without undue reservation.

## Author Contributions

BY, YS, and CM contributed to the conception and design of this research. CM performed the analysis. YS helped to perform the analysis with constructive discussions. BY wrote the first draft of the manuscript. All authors contributed to the manuscript revision, read, and approved the submitted version.

## Funding

This research was supported by the National Science Foundation of China (Grant Nos. 71872152, 71972065, and 72110107002) and the Fundamental Research Funds for the Central University (Grant No. SWU2209241).

## Conflict of Interest

The authors declare that the research was conducted in the absence of any commercial or financial relationships that could be construed as a potential conflict of interest.

## Publisher’s Note

All claims expressed in this article are solely those of the authors and do not necessarily represent those of their affiliated organizations, or those of the publisher, the editors and the reviewers. Any product that may be evaluated in this article, or claim that may be made by its manufacturer, is not guaranteed or endorsed by the publisher.

## References

[ref1] Aiken L. S. West S. G. (1991). Multiple Regression: Testing and Interpreting Interactions. Newbury Park, CA: Sage.

[ref2] Bahadur W. Ali A. (2021). Linking leader humility with service performance: the role of service climate and customer mistreatment. Asian Bus. Manag. 1–22. doi: 10.1057/s41291-020-00145-9

[ref3] Bass B. M. (1998). “The ethics of transformational leadership,” in Ethics, The Heart of Leadership. ed. Ciulla J. B. (Westport CT: Quorum Books), 169–189.

[ref4] Bauer D. J. Preacher K. J. Gil K. M. (2006). Conceptualizing and testing random indirect effects and moderated mediation in multilevel models: new procedures and recommendations. Psychol. Methods 11, 142–163. doi: 10.1037/1082-989X.11.2.142, PMID: 16784335

[ref5] Bharanitharan K. Chen Z. X. Bahmannia S. Lowe K. B. (2018). Is leader humility a friend or foe, or Both? An attachment theory Lens on leader humility and its contradictory outcomes. J. Bus. Ethics 160, 729–743. doi: 10.1007/s10551-018-3925-z

[ref6] Bian Y. J. (1994). Work and Inequality in Urban China (1st ed., p. 286). New York: State University of New York Press.

[ref7] Bian Y. J. (2006). “Guanxi,” in International Encyclopedia of Economic Sociology. eds. Beckert J. Zafirovski M. (New York, NY: Routledge), 312–314.

[ref8] Blau P. (1964). Exchange and power in social life. New York: Wiley.

[ref9] Brislin R. W. (1980). “Translation and content analysis of oral and written material,” in Handbook of Cross-Cultural Psychology. eds. Triandis H. Berry J. W., vol. 2 (Boston, MA: Allyn and Bacon), 389–444.

[ref10] Carnevale J. B. Huang L. Paterson T. (2019). LMX-differentiation strengthens the prosocial consequences of leader humility: an identification and social exchange perspective. J. Bus. Res. 96, 287–296. doi: 10.1016/j.jbusres.2018.11.048

[ref11] Chan S. C. H. Mak W.-M. (2012). Benevolent leadership and follower performance: the mediating role of leader–member exchange (LMX). Asia Pac. J. Manag. 29, 285–301. doi: 10.1007/s10490-011-9275-3

[ref12] Chen X.-P. Chen C. C. (2004). On the intricacies of the Chinese Guanxi: a process model of Guanxi development. Asia Pac. J. Manag. 21, 305–324. doi: 10.1023/B:APJM.0000036465.19102.d5

[ref001] Chen C. C. Chen X.-P. Huang S. (2013). ChineseGuanxi: An Integrative Review and New Directions for Future Research. Manag. Organ. Rev. 9, 167–207. doi: 10.1111/more.12010

[ref13] Chen C. C. Chen X.-P. (2009). Negative externalities of close guanxi within organizations. Asia Pac. J. Manag. 26, 37–53. doi: 10.1007/s10490-007-9079-7

[ref002] Chen Y. Friedman R. Yu E. Sun F. (2011). Examining the positive and negative effects of guanxi practices: a multi-level analysis of guanxi practices and procedural justice perceptions. Asia Pac. J. Manag. 28, 715–735. doi: 10.1007/s10490-009-9176-x

[ref14] Chen Y. Liu B. Zhang L. Qian S. (2018). Can leader “humility” spark employee “proactivity”? The mediating role of psychological empowerment. Leadersh. Organ. Dev. J. 39, 326–339. doi: 10.1108/lodj-10-2017-0307

[ref16] Chen C.-Y. Yang C.-F. (2012). The impact of spiritual leadership on organizational citizenship behavior: a multi-sample analysis. J. Bus. Ethics 105, 107–114. doi: 10.1007/s10551-011-0953-3

[ref17] Cho J. Schilpzand P. Huang L. Paterson T. (2020). How and when humble leadership facilitates employee job performance: the roles of feeling trusted and job autonomy. J. Leadersh. Organ. Stud. 28, 169–184. doi: 10.1177/1548051820979634

[ref18] Derue D. S. Nahrgang J. D. Wellman N. E. D. Humphrey S. E. (2011). Trait and behavioral theories of leadership: an integration and Meta-analytic test of their relative validity. Pers. Psychol. 64, 7–52. doi: 10.1111/j.1744-6570.2010.01201.x

[ref19] Diao H. Song L. J. Wang Y. Zhong J. (2019). Being passionate to perform: the joint effect of leader humility and follower humility. Front. Psychol. 10:1059. doi: 10.3389/fpsyg.2019.01059, PMID: 31139117 PMC6527839

[ref20] Duan J. Guo Z. Brinsfield C. (2020). Does leader integrity facilitate employee voice? A moderated mediation model of perceived risk and leader consultation. Leadersh. Organ. Dev. J. 41, 1069–1087. doi: 10.1108/lodj-08-2019-0353

[ref21] Farh J.-L. Cheng B.-S. (1997). Modesty bias in self-rating in Taiwan: impact of item wording, modesty value, and self-esteem. Chin. J. Psychol. 39, 103–118.

[ref22] Fiske S. T. Taylor S. E. (2020). Social cognition evolves: illustrations from our work on intergroup Bias and on healthy adaptation. Psicothema 32, 291–297. doi: 10.7334/psicothema2020.197, PMID: 32711662

[ref003] Gardner W. L. Karam E. P. Tribble L. L. Cogliser C. C. (2019). The missing link? Implications of internal, external, and relational attribution combinations for leader–member exchange, relationship work, self‐work, and conflict. J. Organ. Behav. 40, 554–569. doi: 10.1002/job.2349

[ref23] Gerstner C. R. Day D. V. (1997). Meta-analytic review of leader-member exchange theory: correlates and construct issues. J. Appl. Psychol. 82, 827–844. doi: 10.1037/0021-9010.82.6.827

[ref24] Hamilton D. L. Grubb P. D. Acorn D. A. Trolier T. K. Carpenter S. (1990). Attribution difficulty and memory for attribution-relevant information. J. Pers. Soc. Psychol. 59, 891–898. doi: 10.1037/0022-3514.59.5.891, PMID: 2266483

[ref25] Hewlin P. F. Dumas T. L. Burnett M. F. (2017). To Thine own self be true? Facades of conformity, values incongruence, and the moderating impact of leader integrity. Acad. Manage. J. 60, 178–199. doi: 10.5465/amj.2013.0404

[ref26] Homans G. C. (1958). Social behavior as exchange. Am. J. Sociol. 63, 597–606. doi: 10.1086/222355

[ref27] Hu J. Erdogan B. Jiang K. Bauer T. N. Liu S. (2018). Leader humility and team creativity: the role of team information sharing, psychological safety, and power distance. J. Appl. Psychol. 103, 313–323. doi: 10.1037/apl0000277, PMID: 29094959

[ref28] Hui C. Graen G. (1997). Guanxi and professional leadership in contemporary Sino-American joint ventures in mainland China. Leadersh. Q. 8, 451–465. doi: 10.1016/S1048-9843(97)90024-2

[ref29] Hui C. Law K. S. Chen Z. X. (1999). A structural equation model of the effects of negative affectivity, leader-member exchange, and perceived job mobility on in-role and extra-role performance: a Chinese case. Organ. Behav. Hum. Decis. Process. 77, 3–21. doi: 10.1006/obhd.1998.2812, PMID: 9924139

[ref30] Jeung C.-W. Yoon H. J. (2018). When leadership elicits voice: evidence for a mediated moderation model. J. Manag. Organ. 24, 40–61. doi: 10.1017/jmo.2017.42

[ref31] Jiang J. Y. Law K. S. Sun J. J. M. (2014). Leader-member relationship and burnout: the moderating role of leader integrity. Manag. Organ. Rev. 10, 223–247. doi: 10.1111/more.12022

[ref32] Kelemen T. K. Matthews S. H. Matthews M. J. Henry S. E. (2022). Humble leadership: a review and synthesis of leader expressed humility. J. Organ. Behav. 1–23. doi: 10.1002/job.2608

[ref33] Kelley H. H. Michela J. L. (1980). Attribution theory and research. Annu. Rev. Psychol. 31, 457–501. doi: 10.1146/annurev.ps.31.020180.002325 20809783

[ref34] Kline R. (2011). Principles and Practice of Structural Equation Modeling (3rd ed.). NY: Guilford.

[ref35] Lam W. Huang X. Snape E. J. A. (2007). Feedback-seeking behavior and leader-member exchange: Do supervisor-attributed motives matter? Acad. Manage. J. 50, 348–363. doi: 10.5465/amj.2007.24634440

[ref36] Lavelock C. R. Worthington E. L. Jr. Griffin B. J. Garthe R. C. Davis D. E. Hook J. N. (2017). “Humility intervention research: a qualitative review,” in Handbook of Humility: Theory, Research, and Applications. eds. Worthington E. L. Jr. Davis D. E. Hook J. N. (New York: Routledge/Taylor & Francis Group), 274–285.

[ref37] Law K. S. Wong C. S. Wang D. X. Wang L. H. (2000). Effect of supervisor-subordinate guanxi on supervisory decisions in China: an empirical investigation. Int. J. Hum. Resour. Manag. 11, 751–765. doi: 10.1080/09585190050075105

[ref38] Li J. Liang Q. Zhang Z. Wang X. (2018). Leader humility and constructive voice behavior in China: a dual process model. Int. J. Manpow. 39, 840–854. doi: 10.1108/ijm-06-2017-0137

[ref39] Liao H. Chuang A. (2007). Transforming service employees and climate: a multilevel, multisource examination of transformational leadership in building long-term service relationships. J. Appl. Psychol. 92, 1006–1019. doi: 10.1037/0021-9010.92.4.1006, PMID: 17638461

[ref40] Liborius P. Kiewitz C. (2022). When leader humility meets follower competitiveness: relationships with follower affective trust, intended and voluntary turnover. J. Vocat. Behav. 135:103719. doi: 10.1016/j.jvb.2022.103719

[ref41] Liu X.-Y. Wang J. (2013). Abusive supervision and organizational citizenship behaviour: is supervisor–subordinate guanxi mediator? Int. J. Hum. Resour. Manag. 24, 1471–1489. doi: 10.1080/09585192.2012.725082

[ref42] Lu Z. Chen A. Song J. J. P. J. (2018). The influence of humble leadership on EMPLOYEES’PROACTIVE behavior—the role of psychological empowerment and conscientiousness. Panyapiwat J. 10, 138–153.

[ref43] Ma C. Wu C.-H. Chen Z. X. Jiang X. Wei W. (2019). Why and when leader humility promotes constructive voice: a crossover of energy perspective. Pers. Rev. 49, 1157–1175. doi: 10.1108/pr-02-2019-0049

[ref44] Mao J. Chiu C. Y. Owens B. P. Brown J. A. Liao J. (2018). Growing followers: exploring the effects of leader humility on follower self-expansion, self-efficacy, and performance. J. Manag. Stud. 56, 343–371. doi: 10.1111/joms.12395

[ref45] Martinko M. J. Harvey P. Douglas S. C. (2007). The role, function, and contribution of attribution theory to leadership: a review. Leadersh. Q. 18, 561–585. doi: 10.1016/j.leaqua.2007.09.004

[ref46] Maslyn J. M. Uhl-Bien M. (2001). Leader-member exchange and its dimensions: effects of self-effort and other's effort on relationship quality. J. Appl. Psychol. 86, 697–708. doi: 10.1037//0021-9010.86.4.697, PMID: 11519653

[ref004] Mayer D. M. Aquino K. Greenbaum R. L. Kuenzi M. (2012). Who displays ethical leadership, and why does it matter? An examination of antecedents and consequences of ethical leadership. Acad. Manag. J. 55, 151–171. doi: 10.5465/amj.2008.0276

[ref47] Mayer R. C. Davis J. H. (1999). The effect of the performance appraisal system on trust for management: a field quasi-experiment. J. Appl. Psychol. 84, 123–136. doi: 10.1037/0021-9010.84.1.123

[ref005] Moorman R. H. Darnold T. C. Priesemuth M. Dunn C. P. (2012). Toward the Measurement of Perceived Leader Integrity: Introducing a Multidimensional Approach. J. Change Manag. 12, 383–398. doi: 10.1080/14697017.2012.728746

[ref48] Moorman R. H. Darnold T. C. Priesemuth M. (2013). Perceived leader integrity: supporting the construct validity and utility of a multi-dimensional measure in two samples. Leadersh. Q. 24, 427–444. doi: 10.1016/j.leaqua.2013.02.003

[ref49] Moorman R. H. Grover S. L. (2009). Why does leader integrity matter to followers? An uncertainty management-based explanation. Int. J. Leadersh. Stud. 5, 102–114.

[ref50] Morris J. A. Brotheridge C. M. Urbanski J. C. (2005). Bringing humility to leadership: antecedents and consequences of leader humility. Hum. Relat. 58, 1323–1350. doi: 10.1177/0018726705059929

[ref51] Muthén L. K. Muthén B. O. (2017). Mplus User’s Guide. Eighth Edition. Los Angeles, CA: Muthén & Muthén

[ref52] Naseer S. Syed F. Nauman S. Fatima T. Jameel I. Riaz N. (2020). Understanding how leaders' humility promotes followers' emotions and ethical behaviors: workplace spirituality as a mediator. J. Posit. Psychol. 15, 407–419. doi: 10.1080/17439760.2019.1615103

[ref53] Ng T. W. H. Feldman D. C. (2010). Organizational tenure and job performance. J. Manag. 36, 1220–1250. doi: 10.1177/0149206309359809

[ref006] Nielsen R. Marrone J. A. Ferraro H. S. (2013). Leading With Humility. New York, NY: Routledge.

[ref54] Nielsen R. Marrone J. A. Slay H. S. (2010). A new look at humility: exploring the humility concept and its role in socialized charismatic leadership. J. Leadersh. Organ. Stud. 17, 33–43. doi: 10.1177/1548051809350892

[ref55] Ou A. Y. Tsui A. S. Kinicki A. J. Waldman D. A. Xiao Z. Song L. J. (2014). Humble chief executive officers’ connections to top management team integration and middle managers’ responses. Adm. Sci. Q. 59, 34–72. doi: 10.1177/0001839213520131

[ref56] Owens B. P. Baker W. E. Sumpter D. M. Cameron K. S. (2016). Relational energy at work: implications for job engagement and job performance. J. Appl. Psychol. 101, 35–49. doi: 10.1037/apl0000032, PMID: 26098165

[ref57] Owens B. P. Hekman D. R. (2012). Modeling how to grow: an inductive examination of humble leader behaviors, contingencies, and outcomes. Acad. Manage. J. 55, 787–818. doi: 10.5465/amj.2010.0441

[ref58] Owens B. P. Johnson M. D. Mitchell T. R. (2013). Expressed humility in organizations: implications for performance, teams, and leadership. Organ. Sci. 24, 1517–1538. doi: 10.1287/orsc.1120.0795

[ref59] Owens B. P. Rowatt W. C. Wilkins A. L. J. H. (2011). “Exploring the relevance and implications of humility in organizations,” in Handbook of Positive Organizational Scholarship. eds. Spreitzer G. M. Cameron K. S. (New York: Oxford University Press), 260–272.

[ref60] Podsakoff P. M. MacKenzie S. B. Podsakoff N. P. (2012). Sources of method bias in social science research and recommendations on how to control it. Annu. Rev. Psychol. 63, 539–569. doi: 10.1146/annurev-psych-120710-100452, PMID: 21838546

[ref61] Preacher K. J. Selig J. P. (2012). Advantages of Monte Carlo confidence intervals for indirect effects. Commun. Methods Meas. 6, 77–98. doi: 10.1080/19312458.2012.679848

[ref62] Qian S. Liu Y. Chen Y. (2020). Leader humility as a predictor of employees’ feedback-seeking behavior: the intervening role of psychological safety and job insecurity. Curr. Psychol. 41, 1348–1360. doi: 10.1007/s12144-020-00663-x

[ref63] Qin X. Chen C. Yam K. C. Huang M. Ju D. (2020). The double-edged sword of leader humility: investigating when and why leader humility promotes versus inhibits subordinate deviance. J. Appl. Psychol. 105, 693–712. doi: 10.1037/apl0000456, PMID: 31670527

[ref64] Rego A. Owens B. Leal S. Melo A. I. Cunha M. P. Gonçalves L.. (2017). How leader humility helps teams to be humbler, psychologically stronger, and more effective: a moderated mediation model. Leadersh. Q. 28, 639–658. doi: 10.1016/j.leaqua.2017.02.002

[ref65] Riaz S. Xu Y. Hussain S. (2018). Understanding employee innovative behavior and thriving at work: a Chinese perspective. Admin. Sci. 8:46. doi: 10.3390/admsci8030046

[ref66] Ryan D. S. (1983). Self-esteem: an operational definition and ethical analysis. J. Psychol. Theol. 11, 295–302. doi: 10.1177/009164718301100402

[ref009] Selig J. P. Preacher K. J. (2008). Monte Carlo method for assessing mediation: an interactive tool for creating confidence intervals for indirect effects [Computer software]. Available at: http://quantpsy.org/medmc/medmc.htm

[ref67] Settoon R. P. Bennett N. Liden R. C. (1996). Social exchange in organizations: perceived organizational support, leader–member exchange, and employee reciprocity. J. Appl. Psychol. 81, 219–227. doi: 10.1037/0021-9010.81.3.219

[ref68] Simons T. Friedman R. Liu L. A. McLean Parks J. (2007). Racial differences in sensitivity to behavioral integrity: attitudinal consequences, in-group effects, and “trickle down” among black and non-black employees. J. Appl. Psychol. 92, 650–665. doi: 10.1037/0021-9010.92.3.650, PMID: 17484548

[ref69] Sousa-Lima M. Michel J. W. Caetano A. (2013). Clarifying the importance of trust in organizations as a component of effective work relationships. J. Appl. Soc. Psychol. 43, 418–427. doi: 10.1111/j.1559-1816.2013.01012.x

[ref70] Vera D. Rodriguez-Lopez A. (2004). Strategic Virtues. Organ. Dyn. 33, 393–408. doi: 10.1016/j.orgdyn.2004.09.006

[ref71] Verhezen P. (2008). “Guanxi: networks or nepotism?” in Europe–Asia Dialogue on Business Spirituality, Antwerp. ed. Zsolnai L. (Apeldoorn: Garant), 89–106.

[ref72] Wang Y. Luo W. Zhang J. Guo Y. (2019). More humility, less counterproductive work behaviors? The role of interpersonal justice and trust. Front. Bus. Res. China 13, 431–448. doi: 10.1186/s11782-019-0069-7

[ref73] Wang L. Owens B. P. Li J. J. Shi L. (2018). Exploring the affective impact, boundary conditions, and antecedents of leader humility. J. Appl. Psychol. 103, 1019–1038. doi: 10.1037/apl0000314, PMID: 29781636

[ref74] Weiner B. (1985). An attributional theory of achievement motivation and emotion. Psychol. Rev. 92, 548–573. doi: 10.1037/0033-295x.92.4.548, PMID: 3903815

[ref007] Yang K. S. (1992). “Chinese social orientation: From the social interaction perspective” in Chinese Psychology and Behaviour. eds. Yang K. S. Yu A. B. (Laurel, Taipei: Kew Gong Book), 87–142.

[ref008] Yang F. Liu J. Wang Z. Zhang Y. (2017). Feeling energized: a multilevel model of spiritual leadership, leader integrity, relational energy, and job performance. J. Bus. Ethics 158, 983–997. doi: 10.1007/s10551-017-3713-1

[ref75] Yu L. Duffy M. K. (2021). The whiplash effect: the (moderating) role of attributed motives in emotional and behavioral reactions to abusive supervision. J. Appl. Psychol. 106, 754–773. doi: 10.1037/apl0000810, PMID: 32673027

[ref76] Zhai Q. Lindorff M. Cooper B. (2013). Workplace Guanxi: its dispositional antecedents and mediating role in the affectivity–job satisfaction relationship. J. Bus. Ethics 117, 541–551. doi: 10.1007/s10551-012-1544-7

[ref77] Zhang L. Deng Y. Wang Q. (2014). An exploratory study of Chinese motives for building supervisor–subordinate Guanxi. J. Bus. Ethics 124, 659–675. doi: 10.1007/s10551-013-1899-4

[ref78] Zhang L. Lam C. F. Deng Y. (2017). Leader–member exchange and guanxi are not the same: differential impact of dyadic relationships on fit perceptions, helping behavior, and turnover intention. Int. J. Hum. Resour. Manag. 28, 1005–1030. doi: 10.1080/09585192.2015.1128469

[ref79] Zhang Z. Song P. (2020). Multi-level effects of humble leadership on employees’ work well-being: the roles of psychological safety and error management climate. Front. Psychol. 11:571840. doi: 10.3389/fpsyg.2020.571840, PMID: 33262726 PMC7685992

[ref80] Zhu Y. Zhang S. Shen Y. (2019). Humble leadership and employee resilience: exploring the mediating mechanism of work-related promotion focus and perceived insider identity. Front. Psychol. 10:673. doi: 10.3389/fpsyg.2019.00673, PMID: 31001166 PMC6456677

